# Cells in Silico – introducing a high-performance framework for large-scale tissue modeling

**DOI:** 10.1186/s12859-020-03728-7

**Published:** 2020-10-06

**Authors:** Marco Berghoff, Jakob Rosenbauer, Felix Hoffmann, Alexander Schug

**Affiliations:** 1 Steinbuch Centre for Computing, Karlsruhe Institute of Technology, Eggenstein-Leopoldshafen, 76344 Germany; 2 John von Neumann Institute for Computing, Jülich Supercomputer Centre, Forschungszentrum Jülich, Jülich, 52428 Germany; 3 Faculty of Biology, University of Duisburg-Essen, Essen, 45141 Germany

**Keywords:** Tissue growth, Massively parallel, Cellular Potts model

## Abstract

**Background:**

Discoveries in cellular dynamics and tissue development constantly reshape our understanding of fundamental biological processes such as embryogenesis, wound-healing, and tumorigenesis. High-quality microscopy data and ever-improving understanding of single-cell effects rapidly accelerate new discoveries. Still, many computational models either describe few cells highly detailed or larger cell ensembles and tissues more coarsely. Here, we connect these two scales in a joint theoretical model.

**Results:**

We developed a highly parallel version of the cellular Potts model that can be flexibly applied and provides an agent-based model driving cellular events. The model can be modular extended to a multi-model simulation on both scales. Based on the NAStJA framework, a scaling implementation running efficiently on high-performance computing systems was realized. We demonstrate independence of bias in our approach as well as excellent scaling behavior.

**Conclusions:**

Our model scales approximately linear beyond 10,000 cores and thus enables the simulation of large-scale three-dimensional tissues only confined by available computational resources. The strict modular design allows arbitrary models to be configured flexibly and enables applications in a wide range of research questions. Cells in Silico (CiS) can be easily molded to different model assumptions and help push computational scientists to expand their simulations to a new area in tissue simulations. As an example we highlight a 1000^3^ voxel-sized cancerous tissue simulation at sub-cellular resolution.

## Background

The mathematical description of organisms dates back to the beginning of the 20th century [1]. Since then, the theoretical understanding of biology has grown steadily, showing a more and more complex picture. With the emergence of computational models in physics, biophysicists started to adapt those models to describe biological processes [2]. An early development describing tissue development and cell–cell interactions was the so-called cellular Potts model (CPM) by Graner and Glazier ’92 [3]. This model derives from the Potts model and describes cells as connected areas on a grid. They were able to replicate known biological phenomena, such as adhesion driven cell sorting or tissue-growth. From then on, experimental insight into tissue on the cellular level as well as the power of computers has grown steadily, while the size and extent of cell-based tissue simulation have not proportionally evolved. Here, we present a modular framework for supercomputers to accommodate large-scale simulations of tissue with sub-single cell resolution.

### Related work

There are several attempts to parallelize the CPM. Scianna and Preziosi [4] give an overview over advantages and disadvantages. Different methods were applied; for example, shared memory approaches set a lock to the memory that is accessed from parallel processes. Tomeu et al. [5] introduce a lock-free approach: the stencils compute concurrently, the write-back is only allowed if there are no other changes on the specific data, else an unroll is done. Some authors replace the random sampling of the field in the Monte Carlo, with a random walker that is simpler to parallelize. Gusatto et al. [6] used a mutex for shared memory and Cercato et al. [7] used a distributed memory version. Those implementations provide a maximum speedup of 5.4 for 12 cores and a decreasing speedup for increasing core numbers.

Another method that works for shared and distributed memory is a checkerboard method introduced by Chen et al. [8, 9] Here, the distributed sub-domains are split into 2×2×2 parts, and only one part is active so that there is no overlap with other processes. For this model, a trade-off between accuracy and speed has been observed. If the sub-domain part is changed with a high frequency, a lot of communication is done compared to the runtime. On the other side, if it changes with a low frequency, cell movements stick to sub-domain boundaries. Tapia and D’Souza [10, 11] use this method to implement a single Graphics Processing Unit (GPU) version. Yu and Yang [12] use OpenCL to execute their model on GPUs and multi-core Compute Processing Units (CPUs).

He et al. [13] present a hybrid parallel version, where the CPM is calculated in shared memory, while additional partial differential equations use distributed memory methods.

## Implementation

Cells in Silico (CiS), was implemented into the NAStJA (Neoteric Autonomous Stencil code for Jolly Algorithms) framework [14, 15]. Implementing the parallel CPM into the framework imposed several challenges, such as quasi global cell-state information and isotropic sampling of the field. To incorporate all necessary prerequisites, the framework was vastly extended to provide all the required infrastructure for large scale tissue simulations with the CPM.

### NAStJA framework

**Fig. 1:**
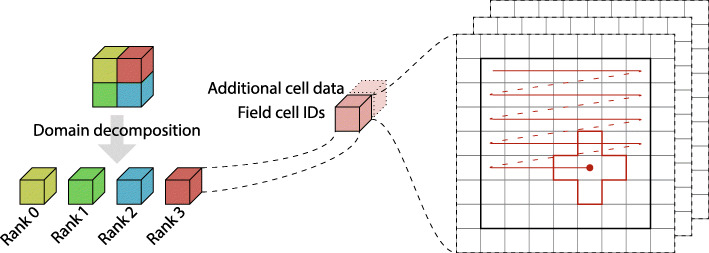
The domain is decomposed and distributed to MPI ranks. So each rank holds one block. Each block contains a field with the cellID and additional cell data. A field is a three-dimensional array on which the compute actions are performed

The NAStJA framework is a modular, flexible framework for massively parallel stencil code applications. It uses the Message Passing Interface (MPI) to communicate between processes. The entire simulation domain is decomposed into small blocks, and these blocks are distributed to the different MPI ranks, see Fig. 1.

Blocks represent a skeleton of the geometry, i.e., the size and position of the parallel entity in the global domain. Stencil codes act on regular grids, called fields. The blocks can hold one or more fields. The data inside the fields are located in voxels. Each voxel contains a data value. For the calculation, a stencil containing the calculation rules is applied to the voxels of the field. The calculation rule determines the data access pattern of the stencil. For *n* dimensions with *m* neighbor accesses, the stencil is denoted D*n*C*m*. In three dimensions, usual access patterns are the D3C7, i.e., the central voxel plus the first six neighboring voxels, or the D3C27 with 26 neighboring voxels, i.e., the full 3×3×3 surrounding of a voxel. The neighbors are accessed read-only. Writing is always done at the central voxel of the stencil. For a consistent parallel calculation, the field in each block is enlarged by a halo layer, which overlaps with the neighboring blocks’ fields. In order to keep the data in the halo up-to-date, each time-step is proceeded by a halo exchange.

**Fig. 2:**
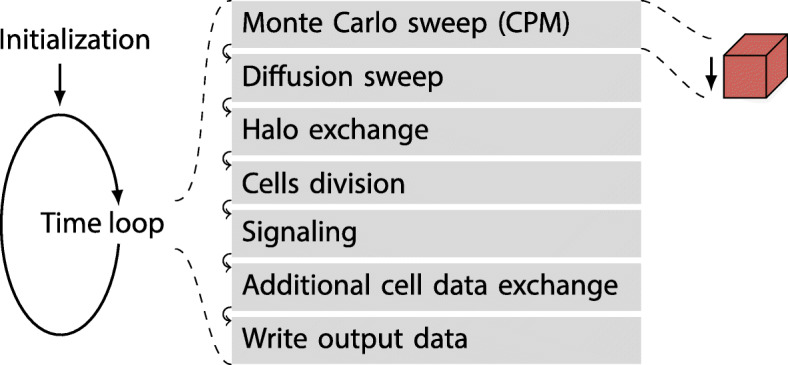
The actions in one time loop are structures in modules. Additional modules can be added depending on the simulated system

After an initialization phase, NAStJA continuously runs the calculation loop. On a time-step base, a sequence of actions is executed independently on each MPI rank. Figure 2 provides an overview of the actions used in CiS. Details to the actions will be given in later sections.

Actions that iterate a stencil over the field are called sweeps. After all sweeps and actions, synchronization steps such as the halo exchange as well as output actions are executed. All sweeps and actions are implemented in a modular fashion, thereby enabling a quick alteration of the code structure by exchanging the modules or changing their order.

Additionally, NAStJA offers an interface for reading in configurations to parametrize simulations. Get-functions are implemented that read the data for a certain config-key from a JSON config file. This allows users to easily parametrize the simulation by changing the config file without modifying and recompiling the code.

### Parallel cellular Potts model

In the last section, we reviewed the framework, the basic structure with blocks and fields, and the flexibility of the actions. This section first describes our implementation of the CPM and then its parallelization.

Each voxel in the field holds an integer value that denotes a cell identifier (cellID). Voxels that contain the same cellID belong to an individual biological cell. In addition to this spatial cell description, each cell has a set of Additional Cell Data (ACD), e.g., the cell volume *V*, the cell surface *S*, the cell age *θ*, and the cell type *τ* (cf. Table 1).

**Table 1:** Cell Properties (dynamically change during simulation)

**Global variables (kept up to date in all blocks)**	
cellID	The Value in the field that identify the cell
Volumes	The cell Volume
Surface	The cell surface (side counting or marching cubes)
Birth	Time of initialization of the cell
Type	The cell type
Center of mass	Center of mass of the cell.
Signal vector	Signal content of each signal within the cell
**Temporary variables (block internal)**	
Cell neighbor surfaces	Shared surfaces with neighboring cells
*Δ*Volume	Volume change during a MCS
*Δ*Surface	Surface change during a MCS
*Δ*Signal	Signal changes during a MCS

#### Cell types

A cell type is assigned to each cell, determining the parametrization and phenotype of that cell. The cell type defines the characteristics of the individual cells, i.e., the target volume *V*_0_ and the target surface *S*_0_ (cf. Table 2). This allows a parametrization of a set of cells instead of specifying the parameters individually. We introduced a subset of cell types that are not participating in the spatio-temporal propagation via the CPM. Those cells are fixed structures that can model blood vessels or the extracellular matrix, termed *solid* in our framework. The particular cell type *liquid* denotes the surroundings of the cells. It acts as a place holder for the growth of cells and describes the medium into which the cells grow.

**Table 2:** Cell type properties (set by config file)

**Parameter**	**Description**
*V*_0_	Target volume
*S*_0_	Target surface
*λ*_*V*_	Volume coupling factor
*λ*_*S*_	Surface coupling factor
*A*_*i*,*j*_	Adhesion coupling matrix
Size change	constant rate of change of *V*_0_ and *S*_0_
Diffusion matrix	Diffusion constant matrix
Signal decay	Signal decay per time-step (relative and absolute)
Constant signal	Has constant signal
Start signal	Cells of this type are initiated with the signal content
**Division**	
Rate	Division rate
Age	Minimum division age
Signal thresholds	Minimum and maximum signal value
Mutation matrix	Probability to mutate to another type
**Cell death**	
Apoptotic cell type	Cell type of the apoptotic cells
Rate	Cell death rate
Age	Minimum cell age
Signal thresholds	Minimum and maximum signal value

#### Hamiltonian

The CPM was introduced by Glazier and Graner [3] in 1992 to simulate adhesion driven cell sorting. It is based on a Potts model that describes integer spin states on a regular lattice, in both two and three dimensions. The temporal propagation of the system is performed by Monte Carlo Sweeps (MCSs) over the field. Nearest neighbor interactions are evaluated by energy functions and are accepted with the Metropolis criterion. A Hamiltonian energy function defines the system energy, denoted as a sum of energy contributions *E*_*i*_, weighted with *λ*_*i*_. It reads, 
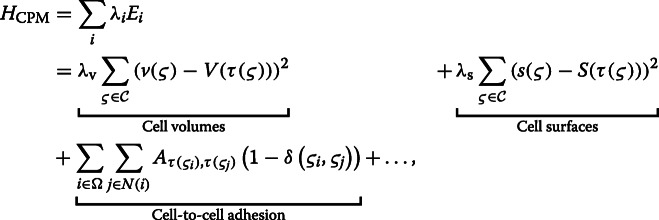
 where $\mathcal {C}$ is the set of all cells, *Ω* is the whole domain, and *N*(*i*) are the neighbors of voxel *i*. Further, *ς*_*i*_ is the corresponding cell at voxel *i*, and *ς*_*j*_ is the corresponding cell at the neighboring voxel. Cell-to-cell adhesion is modeled by an energy contribution that is proportional to the shared surface of different cells. *A* is the adhesion coefficient matrix giving the adhesion between two cells of types *τ*(*ς*_*i*_),*τ*(*ς*_*j*_),*δ* is the Kronecker delta. *v*(*ς*) is the volume of cell *ς*,*V*(*τ*(*ς*)) is the target volume of the cell type, *λ*_v_ is a coupling term regulating the strength of the volume constraint. *s*(*ς*_*i*_) is the surface of cell *ς*,*S*(*τ*(*ς*_*i*_)) is the target surface of the cell type, *λ*_s_ is a coupling term adjusting the strength of the surface constraint. The ellipsis indicates that the energy can be extended with various energy contributions.

The system propagation in the CPM is based on nearest-neighbor interactions. The cellID of a voxel can be changed to the cellID of a randomly chosen nearest neighbor voxel. Then, the energy difference *ΔE* of this local confirmation change is calculated via the change of the Hamiltonian energy function. Changes with negative energy differences are accepted, and positive energy differences have an exponentially decaying acceptance probability

$$\begin{array}{*{20}l} p_{\text{accept}} = \left\{\begin{array}{ll} 1, & \text{if~} \Delta E < 0, \\ \exp(-\Delta E/T), & \text{otherwise}. \end{array}\right. \end{array} $$

This is the Metropolis acceptance criterion with temperature *T*.

#### Energy calculations

The modularity of CiS allows adding various energy functions to the Hamiltonian. Each energy function gets the stencil and the direction of the neighbor as input parameters and returns the energy difference *ΔE* and local change of the surface and volume (*ΔS* and *ΔV*). In the function, the magnitude of the energy is determined by the internal cell states, as well as the corresponding coupling terms *λ*.

**Fig. 3:**
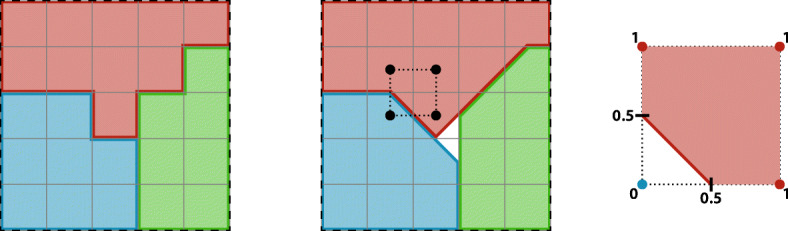
Manhattan surface calculation (left) and a two-dimensional representation of the marching cube surface calculation (middle). With a surface of the red cell 8 using side counting (6.24, marching cubes), blue and green cells 6 (5.12). The marching cubes are shifted at denoted by the black rectangle, i.e., each voxel contributes to four marching cubes in 2D and eight in 3D space. The right side shows a detailed version of one marching cube, determined the surface for the red cell. The edges get the value 1 when it lies inside the red cell, 0 otherwise. The surface then is the 0.5 iso-line

**Surface Calculation** The calculation of the surface of objects on a cubic grid is not unique. Depending on the chosen surface metric, dependencies may occur that prefer some spatial directions, leading to anisotropies in the emerging structures. Traditionally, a Manhattan metric is used to calculate the surface in the CPM. With this metric, the distance *d* between two points ***a***,***b*** is defined by the sum of the absolute differences of their coordinates, $d(\boldsymbol {a},\boldsymbol {b})=\sum _{i} \mid {a_{i} -b_{i}}\mid $. In two dimensions, this corresponds to counting edges of pixels and in the three-dimensional to counting surfaces of voxels. With this metric, a unit circle has the same surface as a unit square. Likewise, in three dimensions, an ideal sphere of diameter *a* corresponds to a cube of edge length *a* after minimizing the surface. Particularly in the three-dimensional case, cell clusters tend to assume a cubic shape, when using the Manhattan distance for the surface calculations, introducing a non-isotropic grid dependence in the model. In order to ensure a more isotropic sampling of the field and to diminish grid artifacts, we use the marching cubes algorithm [16, 17]. The centers of eight adjacent voxels form the edges for the cube of the marching cube algorithm. Then, we distinguish between all edges that have the cellID that surface is calculated and all other cellIDs. Technically, we calculate the iso-surface for 0.5 by set the corners of the calculated cellID to 1 and all others to 0. The surfaces of both algorithms are presented in Fig. 3.

**Volume Calculation** The volume calculation is either done by counting the voxel or using the marching cube algorithm to approximate the volume.

**Adhesion Calculation** The adhesion energy difference is calculated by using the different surface metrics (side counting or marching cubes) to determine the change in shared surfaces between cells. The energy difference is determined by weighting the surface difference with the adhesion coupling matrix *A*.

#### Parallelism

While being propagated in parallel, the entire field has to be consistent. A stencil is needed for the calculation, which writes at the central position while reading from the neighboring voxels. For neighboring voxels located outside of the current block, a copy of the data from neighboring blocks is available due to the halo exchange. The halo data is constant during a Monte Carlo Sweep (MCS), consisting of a certain amount of Monte Carlo steps. To keep the halo data consistent with the neighbor block’s data, the neighbor must not change the values read by the stencil. Therefore, it must be ensured that the neighboring voxels that are read have not been changed. This strategy is essential for all voxels in the halo. Since each voxel requires a uniform chance of sampling, we extend this condition to the entire field. Consequently, all read values within an MCS are from the previous time-step. Hence, the field data read within an MCS is independent of the access order.

To ensure the separation of read and written data, we introduce voxel-wise disjoint subsets similar to the black and white squares on a checkerboard. These subsets are regularly distributed over the entire domain, and only one subset is set active, i.e., only these voxels can be changed during system propagation. Note, the stencil can read all other voxels for the calculations. On the one hand, this ensures a uniform access pattern by not handling the boundary separately. On the other hand, it ensures that a stencil with a white center only reads from black fields. This satisfies the prerequisites described above.

During one MCS, the cell properties, such as surface and volume, stay constant. All changes in those properties are accumulated to delta storages, e.g., *ΔS* and *ΔV*. After one MCS, a synchronization step exchanges the halo and the deltas. The subsequent MCS acts on another active subset.

#### Checkerboards

**Fig. 4:**
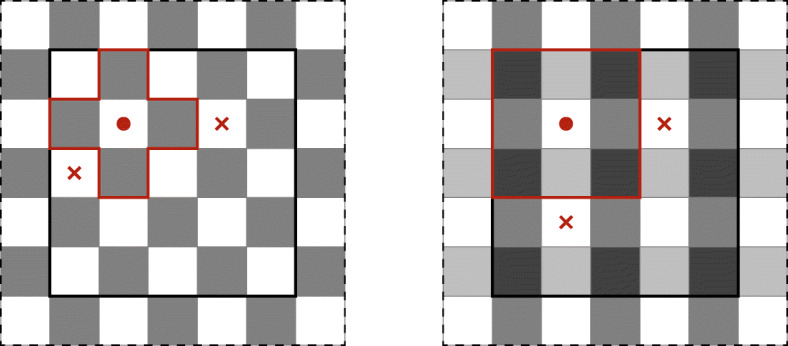
A two-dimension representation of the D3C7- or a D3C27-stencil (A 3D stencil with 7 or 27 active voxels, respectively) with the two-colored (left) and eight-colored (right) checkerboard. The white subset is active and hence read and write accessible, while the other colors are read-only. The red dot represents the actual stencil position, and the red surrounding marks the input voxels. The red crosses mark the next possible stencil position in x- and y-directions

The stencil size determines the number of necessary disjoint subsets. Figure 4 (left) shows a two-dimensional representation of the checkerboard for the D3C7-stencil.

Here, we need two subsets since the stencil only accesses the six nearest neighbors. To stay in the analogy, we denote each subset as a color of the checkerboard. For a given voxel, e.g., the red dot on the white voxel, the direct neighbor voxels are read but are not allowed to change. Diagonal neighbor voxels and the next-nearest neighbor voxels can change, so here a stencil calculation can be performed. In three dimensions, the layer in the front and the back are shifted by color.

Figure 4 (right) presents the eight-colored checkerboard for the D3C27-stencil. The diagonals are used by the stencil itself so that the next stencil can only act on the voxel’s next-nearest neighbors. In three dimensions, the layer in the front and the back use four different colors.

To achieve a uniform probability across the whole field, two or eight MCSs are required for the two- or eight-colored checkerboard, respectively.

#### Quality of pseudo-random numbers

Pseudo-random number generators in parallel applications can produce unintentional patterns [18]. This happens when the sequences overlap in different ranks, and the parallel entities use the same numbers. We use a standard generator based on the Mersenne Twister algorithm. Per MPI rank, one generator is used and initialized based on the MPI rank, so each generator starts on a different position in the random number sequence. We use the generator for all random numbers, e.g., random access and energy acceptance. Depending on the local domain data, a varying amount of random numbers is generated per Monte Carlo step. Therefore blocks with overlapping random number sequences, which is statistically extremely unlikely, do not correlate since the random numbers are used for various purposes.

#### Visitor pattern

We introduce a linear random access pattern. Therefore, the field on the active color is accessed in a linear walk. Since the volumes and surfaces are only updated after a complete MCS, large changes in volume or surface in one sweep produce unwanted behavior since the stored value strongly differs from the actual value. To restrict this discrepancy only a subset of all possible positions is sampled to avoid overshooting the changed parameters. Instead of randomly sample the whole field, we go linearly through the field while skipping a random amount of voxels until the end of the field is reached. By using this linear access pattern cached data can be reused and cache misses can be avoided. We use preliminary virtual voxels to ensure that the first voxel in the sweep is also selected uniformly.

#### Localize global information

**Fig. 5:**
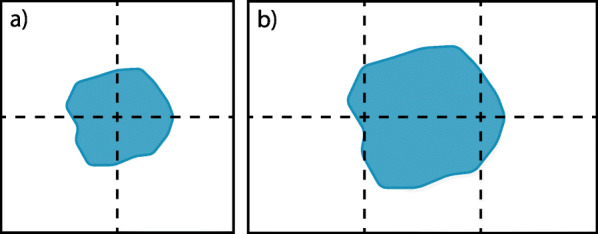
(**a**) Valid and (**b**) invalid distribution of an cell (blue) over blocks (dashed rectangles). The invalid object distribution overlaps three blocks in x-dimension. The size of the cell is larger than the size of one box

The halo exchange ensures the consistency of the field data. Additionally, it must also be ensured that the ACD is updated after each MCS. Each block containing a part of a cell must have up-to-date ACD for that cell. A global exchange using collective MPI functions does not scale very well. However, introducing minor prerequisites allows an exchange of the ACD to all requiring blocks with local communications. If we limit the exchange to the first 26 neighbors, one cell may only stretch beyond the block boundaries on one side per dimension. Consequently, the cell size must be smaller than the block size per dimension, as shown in Fig. 5a. This can be guaranteed if the size of the cells is limited or the block size is large enough.

If a cell is illegitimate large and overlaps three blocks, as shown in Fig. 5b, a ACD exchange will not update consistently in all blocks. The changes in the left blocks do not reach the right blocks and vice versa.

**Fig. 6:**
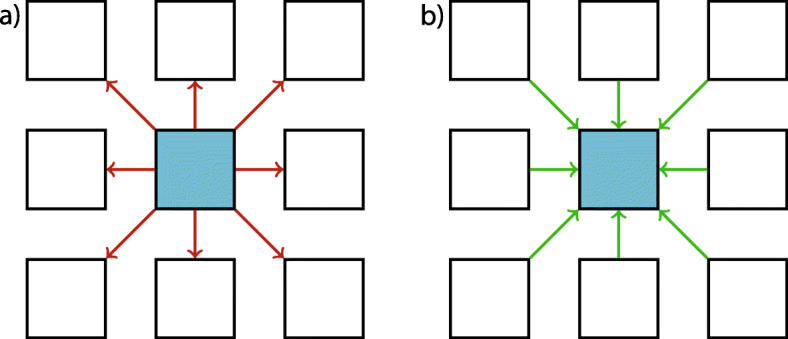
Local neighbor exchange in two dimensions. The center block (blue) (**a**) sends to eight neighboring blocks and (**b**) receives from the same eight neighbors

The exchange is performed after each MCS as shown in Fig. 6.

A message is created that stores the ACD and additional exchanged information. For example, for each cell in a block the values of volume and surface as well as the delta volume and delta surface are stored this message package. Then, the package is sent to all 26 neighbors, received and unpacked. As soon as the data has been received from all neighbors, it can be processed. The sent deltas are accumulated to calculate the absolute values. Since cells move, they can newly enter into blocks. So, in addition to the changes of volume and surface, their absolute values must be transferred, such that the newly entered blocks can calculate the current volume and surface from the changes. The amount of transferred data depends on the number of different cells and the number of different types of values. Details of this exchange can be found in Ref. [19]. Each block holds the ACD for all cells, which are inside the block or in the halo. ACD for cells that are no longer in the block or halo is removed.

### Cell events

Single-cell events have to be processed simultaneously on all blocks that hold a part of the specific cell. Therefore, single-cell events are split into two steps. The first step is the *determination step*, where events are detected and determined. This only happens in one block, namely the block containing the center of mass of the cell. The event is not executed immediately. The instruction is propagated to all adjacent blocks via the ACD exchange. In the following time-step, the *execution step* is processed in all blocks containing the cell. Here, the single-cell event is then executed consistently across all involved blocks.

### Sanity action

As described above, the efficient parallel calculation requires a restriction of the cell size. Since we cannot have absolute control over every cell via the stochastic process, some single voxels may detach from a cell. Some references prevent this non-biological behavior directly in the energy calculation [20]. Here, we detect single voxels of a cellID without direct contact and replace them with liquid. In the rare event that several connected voxels detach, the identification of a segment is complex and cannot be calculated locally. In this case, we have two options, (i) we delete all voxels outside a predefined radius around the cell center, or (ii) we ignore the voxel detachment as long as it does not violate the requirements. I.e., if the cell and the voxel segment are moving away from each other, the condition that a cell can only go beyond one block boundary per dimension can be violated, which in turn leads to inconsistencies. This is detected within the ACD exchange, and the premature death of the cell will be inaugurated. If an ACD exchange receives ACD for one cell from opposite sides, then a so-called Message of Death (MoD) will be generated. This MoD is sent for two time-steps to all 26 neighbors, stored and forwarded to the neighbors of the neighbors. And finally, the cell is deleted simultaneously from all 125 neighbor and next-nearest neighbor blocks. This ensures that the cell is deleted from all blocks in which it can occur, and resolve the inconsistencies.

### Agent-based cell actions

In addition to the system propagation described by the CPM, CIS provides several modules that allow multi-model simulation of more complex systems. These modules are using NAStJA’s action system and are implemented as actions acting on the cell objects directly. Cell attributes such as the cell age, the signal level, cell type, etc. are append to the ACD. Actions act depending on these values.

#### Signal and nutrient transport

The simulation considers the transmission and propagation of multiple substances, such as nutrients and drugs. We define a class of signaling, e.g., nutrient contents, of each cell $\sigma ^{(\varrho)}_{i}$, denoting the concentration of signal *ϱ* in cell *i*. Those represent nutrients for the cell (e.g., oxygen, glucose, or an effective nutrient concentration), cell-to-cell signaling compounds, or arbitrary drugs. Diffusion is approximated by a flow through the cells’ surfaces.

**Diffusion** The diffusion of signals between the cells occurs through the surface of these cells. We determine the shared surface *S*_*i*,*j*_ for each pair of cells *i*,*j* with *i*≠*j*. The shared surfaces of cells are determined in a sweep over the field that locally saves the neighbors as well as the respective shared surface of each cell. The diffusion depends on the type of cells, so we define for each combination of types a diffusion constant *D*_*τ*(*i*),*τ*(*j*)_,*τ*(*i*) denoting the cell type of cell *i*. The flux $J_{i,j}^{(\varrho)}$ for a signal *ϱ* is defined by

$$\begin{array}{*{20}l} J_{i,j}^{(\varrho)} = \left(\frac{S_{i,j}}{S_{i}} + \frac{S_{i,j}}{S_{j}}\right) D_{\tau(i),\tau(j)} \left(\sigma_{j}^{(\varrho)} - \sigma_{i}^{(\varrho)}\right), \end{array} $$

where *S*_*i*_ is the surface from cell *i* and *σ*_*i*_ is the signal value in cell *i* and *S*_*j*_,*σ*_*j*_ from cell *j*, respectively. This is the arithmetic mean of the two surface fractions with respect to the common surface. The flux *J*_*i*,*j*_ is subtracted from the delta signal of one cell and added to the other. Here, we distinguish between cells and fixed signal suppliers, such as blood vessels. For fixed signal suppliers, the signal content is kept constant, i.e., the flux is neither subtracted nor added for those cells. In order to keep the signal contents of all cells up to date, the delta signals are communicated with the ACD exchange to all neighboring blocks.

**Decay** Metabolic processes take place inside the cells, those as well as other signal depleting processes are described by the signal decay. In our model, the signals are changed relative to their value,

$$\begin{array}{*{20}l} \left.\sigma_{i}^{(\varrho)}\right|_{\mathrm{t}+1} = d^{(\varrho)}_{\tau(i)} \cdot \left.\sigma_{i}^{(\varrho)}\right|_{\mathrm{t}}, \end{array} $$

where $\left.\sigma _{i}^{(\varrho)}\right |_{\mathrm {t}}$ is the signal *ϱ* in cell *i* at time *t* and $d^{(\varrho)}_{\tau (i)}$ is the relative change of the signal *ϱ* depending on the type of cell *i*.

#### Division and mutation

Cell division is a fundamental property of tissue development. During a cell division, one mother cell splits into two daughter cells. Those daughter cells usually inherit the properties of the mother cell, but in special cases such as asymmetric cell division and mutations, the properties can differ.

In each time-step, each cell is checked for cell-division. Whether a cell divides depends on several internal and external factors. Division happen with the division rate *R*_Div_ when the following conditions are fulfilled:

- Volume above a threshold *V*>*V*_Div_=0.9·*V*_0_.
- Nutrition above a threshold *C*_DivMin_.
- Age above a certain threshold Age_DivMin_.

Then, a random plane through the cell center as well as a new cell type (see “§Mutation” section) is chosen. To ensure synchronous execution, this decision is then communicated to all neighboring blocks as described in “§Cell events” section. In the next time-step, the cell is split along the previously determined plane. The cell is split, and the two arising cells are reinitialized while measuring surface and volume. One keeps the cellID of the mother cell while the other receives a new cellID. After a cell division, the cellular age is set to zero for both daughter cells. Post division, both cells expand enforced by the volume and the surface energy term. Specific cell types can also be excluded from cell-division.

##### Mutation

Mutation can occur with a rate of *R*_Mut_, which is defined per cell type. If a mutation is accepted, one of the daughter cells is assigned a randomly selected cell type. A transition matrix between all cell types can be defined so that the transition probabilities between cell types vary. If no mutation occurs, the new cell inherits the type of the mother cell.

#### Cell death

The cell death is implemented with a death rate of *R*_Death_, when the following conditions are fulfilled:

- Nutrition below a threshold *C*_Death_.
- Age above a certain threshold Age_DeathMin_.

Furthermore, cells dying with a reduced death rate *R*_Death_/1000 to account for natural cell death. To ensure simultaneous execution of a cell death across all blocks, the death decision is communicated to all neighboring blocks as described in “§Cell events” section. Cell death is induced by changing the cell type to a dedicated cell type that describes dying cells. For this cell type the target volume in the Hamiltonian is changed over time *V*_0Apop_(*t*)=*V*_0_−*χ*·age, effectively lowering the volume of the cell to zero voxels with a linear temporal dependence on the factor *χ*, that can be set for each cell type. Once the cell reaches *V*=0, the cell and its ACD are deleted.

### Output

NAStJA provides several input and output methods. In the following, we present the relevant writers for CIS. A writer is an action that can prepare, collect, and write out simulation data. The time resolution of the output can be chosen so that every *n* MCS an output frame is created.

#### CellInfo

The CellInfo writer outputs the ACD data of all cells to a comma-separated values (CSV) file per frame. The first line is a header that describes the parameter in each column. Each other row contains the data of one cell, e.g., cell type, age, volume, surface, center of mass, signals. Technically, each worker process creates the output of all cells that center of mass is inside its blocks. A master process collects this and writes it to a file.

#### Parallel VTK

**Fig. 7:**
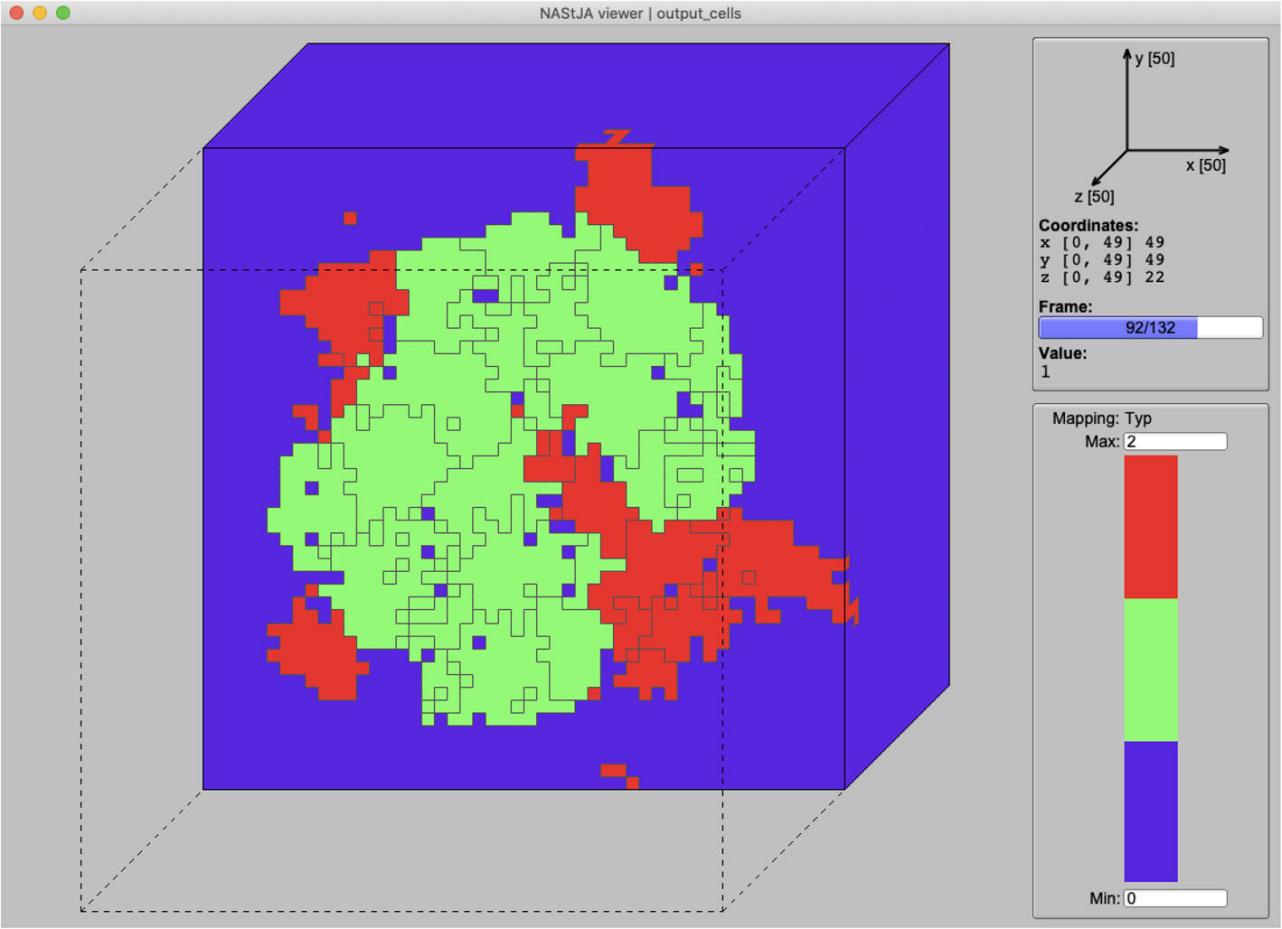
The NAStJA viewer visualizes a slice of the domain. Black outlines mark cells, and the color represents a post-processing value denoting the neighboring to specific cell types

The field data containing the cellIDs is written to a file in parallel using MPI-IO. Resulting in a single binary VTK image (VTI) file per frame. The file contains the whole simulation domain stored in a regular grid similar to a three-dimensional (3D) image. Each value is represented by a 32 bit or a 64 bit integer value, depending on the expected number of total cells. While CSV is a simple text file format, it can be easily read and processed. The VTI files can be read with the Visualization Toolkit (VTK) that provides a python binding and is supported by visualization software like ParaView. These file formats (.csv and.vti) together provide maximum interchangeability with other tools. Furthermore, we developed the NAStJA viewer. A fast and lightweight, quasi 3D visualization software that natively supports the combination of VTI and CSV files, as demonstrated in Fig. 7. It is freely available under https://gitlab.com/nastja/viewer.

## Results and discussion

In this section, we first show that the changes to the traditional model necessary for parallelization do not change the behavior of the model. Then we show the parallel performance and usage.

### Statistical analysis

**Fig. 8:**
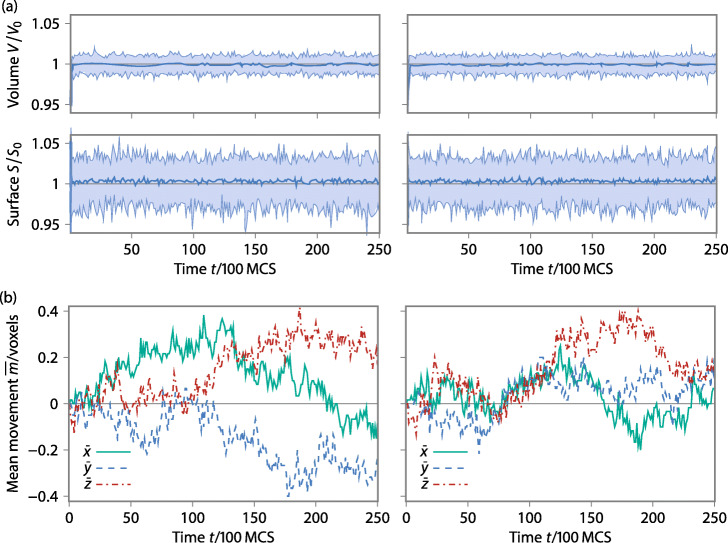
(**a**) Ratio of the surface and volume to the goal values (*V*_0_ and *S*_0_) respectively (averaged over 60 simulations, shaded area indicates standard deviation). In the left plots the cell lies in the center of a block, on the right, the cell overlaps all eight blocks. (**b**) Movement of the cell center in the absence of an external potential. Averaged over 60 simulations

We verified that the execution in our parallelized framework does not distort the model behavior and does not depend on the chosen subdivision. We run 60 simulations with two cells distributed to 2×2×2 blocks. We use cubic blocks, such that a block size of 100 refers to a cubic block with an edge length of 100 voxels without the halo. In the following, we write for cubic blocks shortly 100. The one cell is set to the center of one block and the other cell is set to the edge of all blocks, i.e., to the center of the whole domain. The cells have *V*_0_=1 000 and *S*_0_=1 400. The marching cube algorithm is used for the surface calculations. Figure 8a shows the average over all simulations of the fluctuation in volume and surface over 250 000 MCSs, on the left side, the center cell and on the right the edge cell.

The behavior does not differ depending on the position within the subdivided field, i.e., a cell overlapping two blocks does not experience any directional bias. The temporal variation in surface and volume is statistically around 3%, this is due to the thermal fluctuations introduced by the metropolis criterion as well as a minor contribution of the delayed update of volumes and surfaces. Figure 8b shows the average position of cell center. Note, the cell centers are represented by an integer value denoting a specific voxel. The center of the cell statistically moves around the original position to a very small extent (0.4 voxels) in comparison to the extent of the cell (10 voxels). The movement of the cells also does not depend on the position within the subdivided field. These results confirm the strategy of the parallelization is valid.

### Performance and scaling

We use a single node (kasper) and the high-performance computing systems ForHLR II at the Karlsruhe Institute of Technology (fh2) and JUWELS at the Jülich Supercomputing Centre to perform the performance and scaling tests. The single node has two quad-core Intel Xeon processors E5-2623 v3 with Haswell architecture running at a base frequency of 3 GHz, and have 4 × 256 KB of level 2 cache, and 10 MB of shared level 3 cache. The node has 54 GB main memory.

The ForHLR II has 1152 20-way Intel Xeon compute nodes [21]. Each of these nodes contains two deca-core Intel Xeon processors E5-2660 v3 with Haswell architecture running at a base frequency of 2.6 GHz, and have 10 × 256 KB of level 2 cache, and 25 MB of shared level 3 cache. Each node has 64 GB main memory, and an FDR adapter to connect to the InfiniBand 4X EDR interconnect. In total, 256 nodes can be used, which are connected by a quasi fat-tree topology, with a bandwidth ratio of 10:11 between the switches and leaf switches. The leaf switches connect 23 nodes. The implementation of Open MPI in version 3.1 is used.

JUWELS has 2271 48-way Intel Xeon compute nodes [22]. Each of these nodes contains two 24-core Intel Xeon Platinum 8168 with Skylake architecture running at a base frequency of 2.7 GHz, and have 24 × 1 MB of level 2 cache, and 24 × 1.375 MB of level 3 cache. Each node has 96 GB main memory, and an InfiniBand 4X EDR interconnect. ParaStation MPI in version 5.4 is used.

**Fig. 9:**
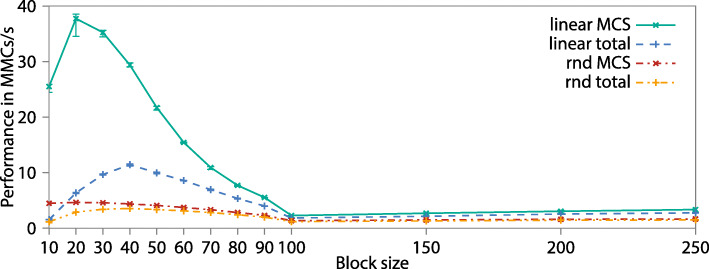
Cache usage and performance of the pure MCS and the total time-step for a linear access pattern with random step width (linear) and a pure random access pattern (rnd)

**Node-level** We run single-core CPM simulations including boundary condition (halo exchange) and the ACD exchange with sending and receiving on the same core. Since the cores in one processor have a shared level 3 cache and we want to avoid the related effects, we run a single-core application on each core simultaneously. Two different access patterns are used, a random access to the active checkerboard color and our linear access using a random jump width. Both methods use a mean voxel step width of 40. We vary the block size and compare the performance of the code in Fig. 9.

As a metric we use the number of million Monte Carlo step attempts per second (MMCs/s). The pure MCS (execution time of CPM-sweep) performance reaches the maximum of 37.8 MMCs/s for 20 blocks with a voxel step width of 40 (yellow line) It decreases until 100 and stays more or less constant for larger block sizes. The peak is clearly defined by the level 2 cache that has a maximum capacity of an equivalent 32 block, but is not exclusively usable by this data. Even if the whole block does not fit into the cache, we can profit from the property of the stencil to access only three layers of the field, the three layers are cached by the level 3 cache, and the data can be reused. The level 3 cache has a capacity of an equivalent of a 68 block, so that until this size no access to the main memory is needed. This describes the slope change in the curve at a block size of 60. The random access pattern can not benefit so much from caching the field and reaches only a peak performance of 4.6 MMCs/s.

The total time-step including exchange and cleaning stages, reaches a peak-performance of 11.4 MMCs/s at a block size of 40 for the linear access pattern, and 3.5 MMCs/s for the random access pattern, respectively. Here, we see that the overlap of the calculation and a nearly constant management overhead shifts the peak to a larger block size.

**Fig. 10:**
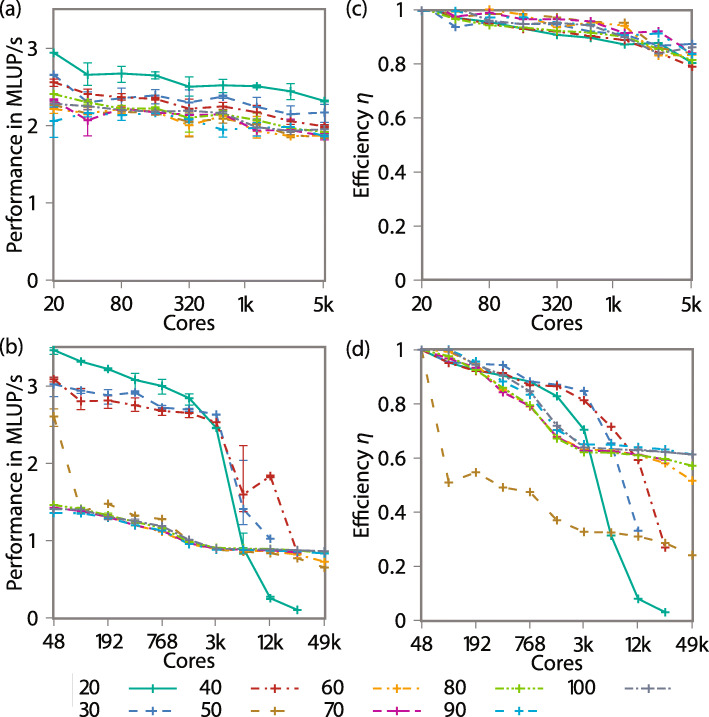
Weak-scaling performance per core on fh2 (**a**) and JUWELS (**b**) in MLUP/s, i.e., scaled by the number of voxels per block. Efficiency of the scaling on fh2 (**c**) and JUWELS (**d**) of the CPM simulations, for the entire time-step, including halo- and ACD-exchange. The error bars denote the slowest and fastest run

**Scaling** For testing the parallel scaling and efficiency, we use weak scaling. The simulation is initialized as a densely filled area of cells with a volume of 512 voxels each. The MCS used a mean step-width of five, with the eight-colored checkerboard. Signal diffusion is enabled. For each core we use one block, the size is varied from 20 to 100. Each simulation runs three-times on 1 to 256 nodes on fh2 and 1024 nodes on JUWELS. The largest simulations are containing approximately 100 million individual cells. Figure 10 shows the scaling performance and efficiency for up to 49 152 cores on JUWELS.

The efficiency *η*=*T*_1_/*T*_*n*_, where *T*_1_ is the reference time for one node and *T*_*n*_ is the time for *n* nodes. On JUWELS (10b) two ranges can be recognized. First the small blocks (20 –40) which show a high performance in the beginning and slow down with many cores, 128 nodes for 20 or 256 nodes for 30 and 40. The large blocks (60 –100) do not reach the maximum performance, but do not drop down much and reach an efficiency of 60% on 49k cores. Up to 128 nodes (6 144 cores), a parallel efficiency of over 85% is reached for the small blocks. For more nodes, the communication overhead becomes significant compared to the calculation time for the small blocks. The gap between the small and large blocks reflects the influence of the cache examined in the previous section. Note that the block size 50 benefits from the cache for one node, but not for two and more nodes. The efficiency based on two nodes would show a scaling similar to the larger blocks.

On the fh2, the performance per core lies between the small and large blocks on JUWELS. Compared to all blocks, only the 20 shows a slightly better performance. The efficiency on 256 nodes (5 120 cores) is 80% – 90%.

### User interface

**Fig. 11:**
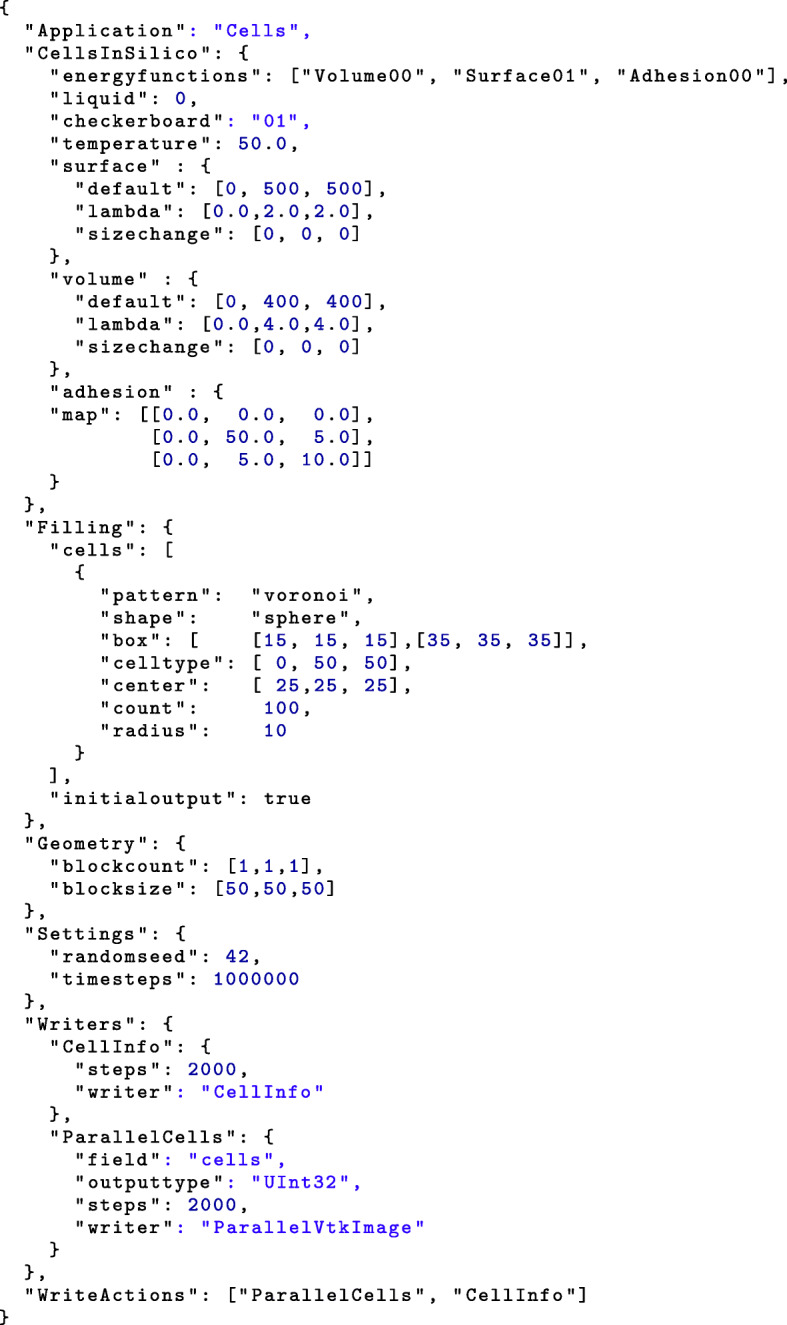
Minimal example of a config for a cell simulation

The model parameters can be flexibly specified through a JSON config file. Multiple field initialization functions allow the placement of cells in the field. The placement of single cells at defined positions, as well as sets of cells in predefined shapes (cubes and spheres), are possible. Figure 11 list a example config file. The result is presented in Fig. 7.

Since the actions, sweeps, and energy functions are modularly designed, it is possible to add specialized and additional code to the system by simply adding a function in a C++ file. Each function is documented in the code and input and output specifications are specified in the doxygen documentation of the code.

Currently, CiS is under heavy development to introduce new Features to discover new effects. Since NAStJA already provides a GPU infrastructure, we plan to provide a multi GPU implementation.

**Fig. 12:**
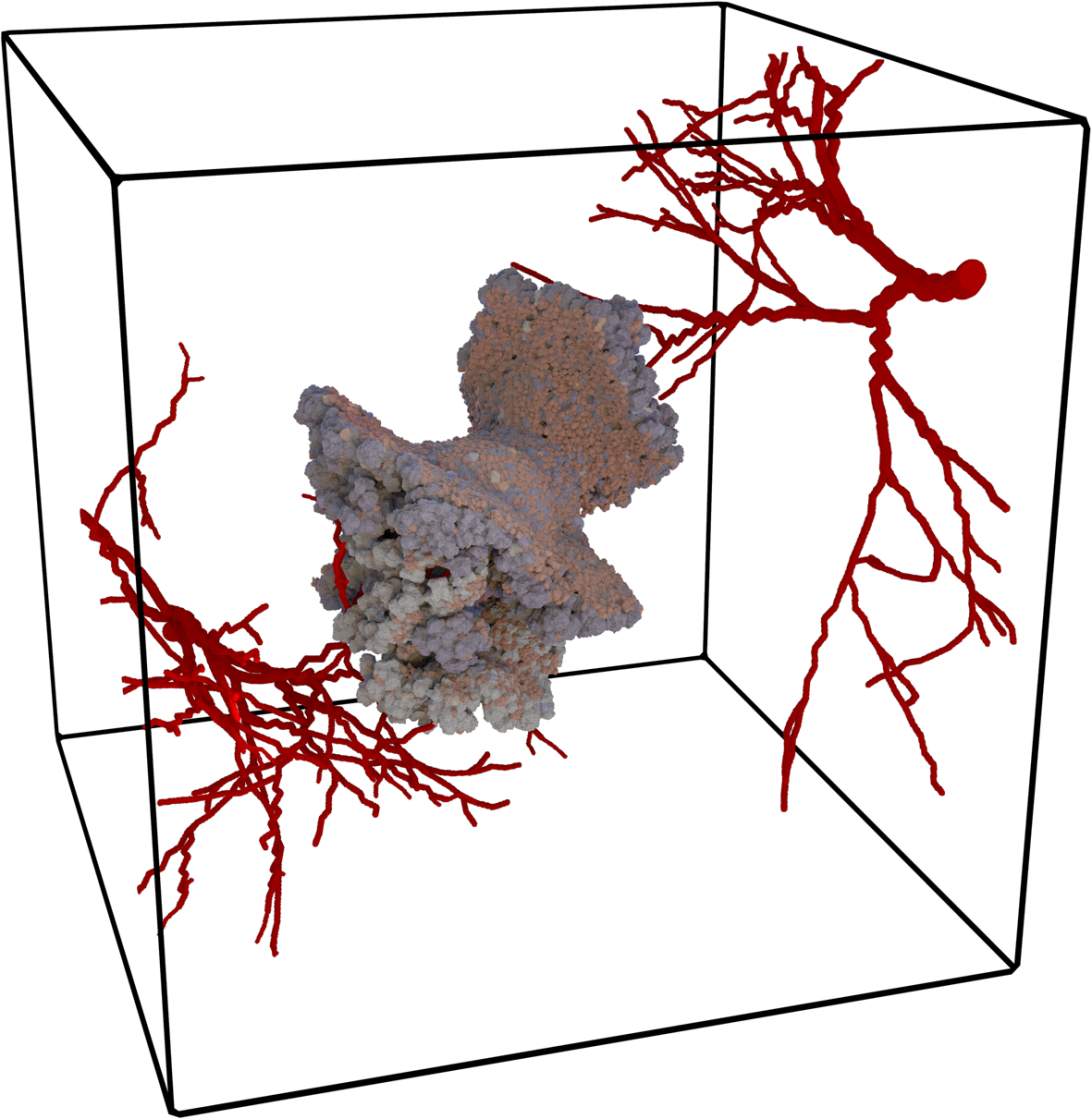
Evolution of a tumor using CiS. Starting from a few tumor cells, the cancer grows along the nutrient-supplying blood vessels. For a clearer view, the surrounding healthy cells are not visible

Without bias on the subdivision and grid, the model enables larger simulated volumes than other implementation of the CPM. These large scales allow us to study, among many other applications, emergent behavior of single-cell shapes to macroscopic tissues as well as tissue scale effects. Figure 12 shows a simple tumor model [23], in which a tumor seed grows through cell divisions into a large tumor.

Cells can acquire new types at cell division, corresponding to mutations in cells that yield new phenotypes. Cell division and cell death, depending on the availability of nutrients, which are distributed from a set of stationary blood vessels. Through the parametrization of a set of phenotypes, tumor development and the emergence and evolution of heterogeneity can be observed and tracked. The simulation yields a trajectory of each individual cell through time, giving access to all properties. Here, the effect of single-cell properties can be observed at a tissue scale. Tissue scale effects, such as fingering growth and a necrotic core, are also observable. Simulations are performed on a field consisting of 1 000×1 000×1 000 voxels and including 10^6^ cells with 1 000 voxels each. The calculation was distributed to 1 000 CPU-cores and simulated for 24 h on the high-performance computing (HPC) system JUWELS [22]. The reached simulated time is around nine months in real-time.

## Conclusions

We introduce a parallel implementation of the cellular Potts model (CPM) and demonstrate that the parallelization is bias-free. Around the CPM we developed the extendable parallel simulation framework Cells in Silico (CiS) for the simulation of tissue growth. CiS provides a user-friendly environment to implement new models. It provides an excellent scalability on supercomputers with a parallel efficiency of up to 90% on a small machine (fh2) with 5 120 cores. Larger simulations show an efficiency of up to over 60% for 49 152 cores, the lower efficiency is explained by an additional layer of switches, which is required for large simulations. The demonstrated performance per core for a full time-step is between 1 and 3.5 MLUP/s depending on the block size and the number of cores. With this scaling behavior, CiS enables large-scale tissue simulations up to some mm’s and millions of interacting cells, while providing a geometric shape resolution of individual cells. Additionally to the geometry resolved cell simulations, we provide an agent-based model running in parallel performing single-cell events such as cell divisions, mutations, and cell death. Signal and nutrient transport through the tissue are enabled by a diffusion and signaling module that interacts with the cell geometries as well as the agent-based model to determine cell events and behavior. The model setup is designed to be user friendly by setting up simulations through a single configuration file that specifies model behavior and initialization. The simulation output is formatted in transferable easy to access data formats for broad compatibility and the use of standard tools. The entire framework is designed to have a fundamentally modular structure for easy model assembly and quick extension. The model is freely available to everyone under an open-source license.

It enables the use of the framework in a wide range of scientific applications opening up new areas for computational research by connecting the scales between single-cell data and tissue data in a single model. This model can be applied in simulations of tumor evolution and heterogeneity simulations, developmental biology, such as tissue patterning. The large scale of the simulations will enable new simulation of epithelial tissue, such as wound healing. In the future, the model can complement wet-lab experiments and testing through enabling large-scale simulations comparable to experimental and medical imaging methods.

With this contribution, we enable a new scale of tissue simulations that connect single-cell data with tissue scale measurements. We lift the barriers for large-scale simulations to a point where the upper bound is determined not by the model but by the parametrization and our imagination. This paves the way to bridge the scales between microbiological findings and medical images.

## Availability and requirements

- Project name: NAStJA – Cells in Silico
- Project repository: https://gitlab.com/nastja/nastja
- Project home page: https://nastja.gitlab.io
- Operating systems: Linux, Mac OS
- Programming language: C++
- Other requirements: MPI, CMake
- License: Mozilla Public License, version 2.0 (MPL2.0)
- Any restrictions to use by non-academics: none

## Data Availability

The source-code and examples are available on the gitlab repository.

## Abbreviations

2D: Two-dimensional
3D: Three-dimensional
ACD: Additional cell data
cellID: Cell identifier
CiS: Cells in silico
CPM: Cellular Potts model
CPU: Compute processing unit
CSV: Comma-separated values
EDR: Enhanced data rate
FDR: Fourteen data rate
GPU: Graphics processing unit
HPC: High-performance computing
JSON: JavaScript object notation
MCS: Monte Carlo sweep
MLUP/s: Million lattice updates per second
MMCs/s: Million Monte Carlo step attempts per second
MoD: Message of death
MPI: Message passing interface
NAStJA: Neoteric autonomous Stencil code for jolly algorithms
OpenCL: Open computing language
VTI: VTK image
VTK: Visualization toolkit
